# Acute Basilar Artery Occlusion Presenting With Convulsive Movements: A Systematic Review

**DOI:** 10.3389/fneur.2021.803618

**Published:** 2022-01-07

**Authors:** Dan Zhang, Yigang Chen, Yonggang Hao, Xingyue Hu, Xudong He

**Affiliations:** ^1^Department of Neurology, Sir Run Run Shaw Hospital, School of Medicine, Zhejiang University, Hangzhou, China; ^2^Department of Neurology, Dushu Lake Hospital Affiliated to Soochow University, Suzhou, China

**Keywords:** acute ischemic stroke, basilar artery occlusion, posterior circulation stroke, convulsion, seizure

## Abstract

**Background and Purpose:** Convulsive seizures related to posterior circulation stroke are considered rare. However, some patients with acute basilar artery occlusion (BAO) can present with convulsive movements. Misdiagnosed as seizures may delay the reperfusion therapy for acute BAO. In this study, we have summarized the clinical features and possible mechanisms of BAO presenting with convulsive movements.

**Methods:** We performed an Institutional Review Board-approved institutional database query from 2015 to 2020 and a literature search of the online database PubMed. Clinical data were collected and analyzed.

**Results:** In total, 14 patients with acute BAO presented with convulsions. There were 10 men and 4 women, with a mean age of 53 (range, 23–77) years. All of these patients had different degrees of impaired consciousness (100.0%, 14/14). Convulsive movements were the initial symptoms in 78.6% (11/14) of patients. Further, 64.3% (9/14) of patients presented with paralysis or cranial nerve abnormalities, and 85.7% (12/14) of patients were treated with reperfusion therapy (thrombolysis, 35.7% [5/14]; endovascular thrombectomy, 64.3% [9/14]). The BAO etiology and mechanism were related to embolism, vessel dissections, and severe stenosis of the right vertebral artery in 57.1% (8/14), 21.4% (3/14), and 7.1% (1/14) of patients, respectively; they were undefined in 14.3% (2/14) of patients. Moreover, 42.9% (6/14) of patients had a 90-day modified Rankin Scale score of 0–2, and the mortality rate was 21.4% (3/14).

**Conclusions:** Acute BAO, especially that related to embolism or vessel dissection, may present with convulsive movements. Acute BAO is a devastating, but treatable disease if diagnosed in time. Considering the possibility of BAO is important when dealing with patients presenting with acute-onset convulsive movements. Prompt diagnosis and reperfusion therapy may help achieve a better prognosis.

## Introduction

Convulsive seizures can occur in acute ischemic stroke, with an incidence of approximately 5% (1). The risk factors include cortical involvement, multifocal areas of ischemia, anterior circulation or temporal lobe stroke, large infarct size, and ischemic-to-hemorrhagic transformation (1, 2). Generally, seizures occur mainly with cortical infarcts in the anterior circulation or supratentorial lesions; however, seizures related to a posterior circulation infarct are considered rare (3). Acute basilar artery occlusion (BAO) is a potentially fatal, but treatable disease. However, its diagnosis may be challenging due to the heterogeneity of symptoms and signs, especially when the presenting symptom is “seizures.” Occasionally, it may be misdiagnosed as a seizure with Todd's palsy, causing a delay in the treatment of ischemic stroke and leading to a poor prognosis. This study aimed to investigate the clinical characteristics and outcomes of acute BAO presenting with convulsive movements.

## Methods

Data for all consecutive patients with BAO were collected from Sir Run Run Shaw Hospital, PR China between January 1, 2015, and September 1, 2020. This study was approved by the Institutional Review Board Committee, which waived the requirement of written informed consent due to the retrospective study design.

**Figure 1:**
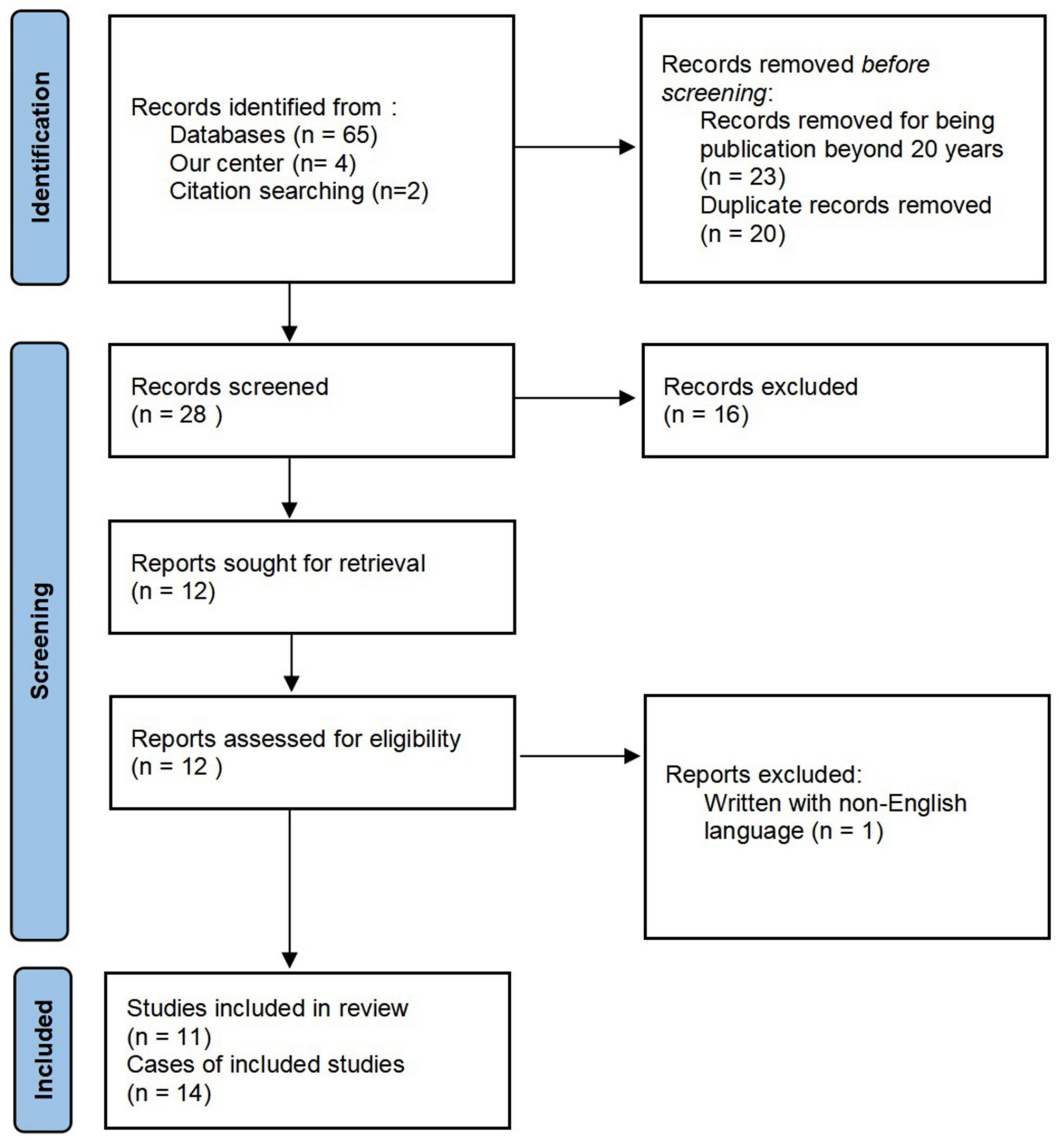
Adopted PRISMA 2020 flow diagram.

In addition, we performed a literature search of the online database PubMed in June 2021 using Endnote-web with the terms “basilar artery occlusion” combined with “seizure,” “convulsion,” “convulsive,” or “epilepsy.” The reference lists of the included articles were screened for additional relevant articles. Considering the development of neuroimaging technology and treatment options, only articles published in the past 20 years were included. Patients with angiography-proven acute BAO, initially presenting with convulsive movements, were included. Asymptomatic patients, patients with chronic BAO, and patients aged <18 years were excluded. Articles without full text available, written in languages other than English, or without sufficient clinical data were excluded. An adapted Preferred Reporting Items for Systematic Reviews and Meta-Analyses (PRISMA) flow diagram is shown in Figure 1.

The full texts of potentially relevant articles were reviewed independently by two authors (DZ and YGC). The following information was extracted from each article: (1) age at onset, (2) sex, (3) initial symptoms and signs, (4) stroke risk factors and comorbidity, (5) infarct regions, (6) result of electroencephalogram (EEG), (7) reperfusion treatment, and (8) prognosis (Table 1).

**Table 1:** Clinical data of 14 acute basilar artery occlusion patients presenting with a convulsive movement.

**Authors, year**	**Age-sex**	**Presenting symptoms and signs**	**EEG**	**Stroke risk factors and comorbidity**	**Acute stage Treatment**	**Infarct region**	**Prognosis**
Saposnik et al. (4)	72 M	Hemiparesis, impaired consciousness, convulsion	No cortical discharge	Hypertension, hypercholesterolemia	AC	Basis and tegmentum of right pons	Poor (dead)
Matsuo et al. (5)	23 M	Loss of consciousness, convulsion–right hemiparesis	Constant diffuse slow, high voltage wave	Basilar artery dissection?	AC	Right cerebellum, left midbrain, bilateral medial temporal lobes, and bilateral thalami	Good
Gadoth et al. (6)	53 M	Vertigo,instability–loss of consciousness, convulsion–right gaze limitation, right hemiplegia	No epileptic activity	Right V4* dissection	EVT	Left cerebellum and pons	Poor (coma)
Wilson et al. (7)	70 F	Impaired consciousness, convulsive movements, right gaze deviation, absent corneal and oculocephalic reflexes	-	AF, hypertension	Thrombolysis	Bilateral cerebellum	Good
Conte et al. (8)	60+ M	Impaired consciousness, convulsive movements, vertical nystagmus	-	Paroxysmal AF	Thrombolysis	Bilateral occipital-temporal lobes, thalami and cerebellum	Poor?
Otsuji et al. (9)	53 M	Convulsion–disturbed consciousness, tetraparesis	-	? (embolus)	EVT	Bilateral cerebellum and thalami, right occipital lobe, left midbrain	Good
	64 M	Convulsion–impaired consciousness, tetraparesis, aphasia	-	Right V4* severe stenosis	EVT	Bilateral cerebellum, right pons, right occipital lobe	Poor (mRS 3)
	77 M	Convulsion–disturbed consciousness, tetraparesis, right homonymous hemianopsia	-	AF	EVT	Left occipital lobe and left lateral posterior choroidal artery	Poor (dead)
Bourmaf et al. (10)	38 M	Dizziness–impaired consciousness, vomiting, convulsion, hyperekplexia	-	Right V1* dissection	EVT	Right pons, left midbrain	Poor (mRS 3)
Bhatt et al. (11)	69 M	Left hand shaking–stuporous, left side convulsion	Epileptic discharge from left frontotemporal region	?	EVT	Bilateral thalami, midbrain, pons, inferior and medial temporal lobes, and occipital lobes	Poor (coma)
Our cases	30 F	Loss of consciousness, convulsion	No epileptic activity	PFO	Thrombolysis	Bilateral midbrain and pons	Good
	35 F	Loss of consciousness, vomiting, convulsive movements	-	PFO	Thrombolysis + EVT	Left pons and cerebellum	Good
	57 F	Loss of consciousness, convulsion, right hemiparesis	-	AF, rheumatic heart disease, valve replacement	EVT	Bilateral pons	Poor (dead)
	45 M	Loss of consciousness, convulsive movements, incontinence	-	PFO	Thrombolysis + EVT	Bilateral cerebellum and midbrain, right thalami and occipital-temporal lobe	Good

*V1 and V4 refer to the 1^st^ and 4^th^ segment of the vertebral artery, respectively. AC, anti-coagulation; AF, atrial fibrillation; EEG, electroencephalogram; EVT, endovascular thrombectomy; mRS, modified Rankin Scale; PFO, patent foramen ovale.

## Results

Over the 5-year study period, 40 patients were diagnosed with BAO at our hospital, among which 28 had acute BAO with new-onset neurological symptoms and signs. Furthermore, 4 patients presented with convulsive movements.

The literature review yielded additional 10 cases of patients with acute BAO presenting with convulsions from eight reports (Table 1). There were 10 men and 4 women, with a mean age of 53 (range, 23–77) years (Table 2). All patients had different degrees of impaired consciousness (100%, 14/14). Further, 78.6% (11/14) of patients initially presented with convulsions or convulsive movements, and 64.3% (9/14) of patients presented with paralysis or cranial nerve abnormalities. However, some patients did not present with focal symptoms, signs, or clear laterality. Electroencephalogram (EEG) results were available for five patients, among which four patients displayed no epileptic activity, and one patient presented with epileptic discharge from the left frontotemporal region.

**Table 2:** Clinical characteristics of acute basilar artery occlusion presenting with convulsions.

	n/N (%)*
Demographics	
Mean age y (SD)	53 (17)
Sex (% of men)	10/14 (71.4%)
Clinical characteristics	
Impaired consciousness (%)	14/14 (100.0%)
Initially presenting with convulsions (%)	11/14 (78.6%)
paralysis or cranial nerve abnormalities (%)	9/14 (64.3%)
Treatment	
Anticoagulation (%)	2/14 (14.3%)
Thrombolysis (%)	5/14 (35.7%)
Endovascular thrombectomy (%)	9/14 (64.3%)
Etiology	
Embolism (%)	8/14 (57.1%)
Atrial fibrillation (%)	4/14 (28.6%)
Patent foramen ovale (%)	3/14 (21.4%)
Artery dissection (%)	3/14 (21.4%)
Vertebral artery stenosis (%)	1/14 (7.1%)
Undefined (%)	2/14 (14.2%)
Prognosis	
mRS 0-2 (%)	6/14 (42.9%)
90-day mortality (%)	3/14 (21.4%)

*Categorical variables are given as n/N, where n is the number of patients in which the variable was present and N is the total number of patients for which that particular variable was reported. mRS, modified Rankin Scale; SD, standard deviation.

In terms of treatment in the hyper-acute stage, all patients, except two patients treated with anticoagulation, received reperfusion therapy (thrombolysis, 35.7% [5/14]; endovascular thrombectomy (EVT), 64.3% [9/14]). Among them, two patients underwent intravenous thrombolysis and bridging artery thrombectomy. Acute endovascular intervention treatment was performed in all patients after 2015. However, EVT was not successful because of a tortuous vertebral artery in one patient (8). In one patient from our center, a 30-year-old woman, digital subtraction angiography was performed after thrombolysis revealed recanalization of the basilar artery.

Regarding the etiology and mechanism of BAO, (1) 57.1% (8/14) of patients had embolism (atrial fibrillation, 28.6% [4/14]; patent foramen ovale, 21.4% [3/14]; unidentified source, 7.1% [1/14]) (9), (2) 21.4% (3/14) of patients had vessel dissections (vertebral artery dissection, 14.3% [2/14]; basilar artery dissection, 7.1% [1/14]), (3) 7.1% (1/14) of patients had basilar occlusion owing to severe right vertebral artery stenosis, and (4) 14.3% (2/14) of patients had an undefined etiology.

Furthermore, 42.9% (6/14) of patients had a 90-day modified Rankin Scale score of 0–2. The all-cause 90-day mortality rate was 21.4% (3/14).

## Discussion

Acute basilar artery occlusion is responsible for approximately 1% of all ischemic strokes, with high rates of mortality and morbidity (12, 13). However, if recognized early, the prognosis may improve with reperfusion therapies such as intravenous thrombolysis and EVT (14, 15). However, in clinical practice, the heterogeneity of presenting symptoms and signs, ranging from a decreased level of consciousness, weakness, cranial nerve abnormalities, and tetraplegia/quadriplegia to locked-in syndrome, can make the diagnosis challenging (7, 16).

Involuntary movements of the limbs are occasionally seen in acute stroke, including fasciculation-like, shivering, jerky, intermittent shaking, and tonic-clonic activities (4). Cortical involvement is the best-characterized risk factor for early seizure after ischemic stroke, which is supported by studies with largely different designs (2, 17). However, seizures related to posterior circulation infarcts are considered rare. According to the cases in our study and the literature, patients with acute BAO may present with convulsions. Moreover, convulsions could even be the initial symptoms in some cases. Patients with acute BAO and pyramidal tract involvement may present with hemiparesis. However, palsy can occur after convulsive seizures, known as Todd's palsy. Therefore, when convulsions are present, especially when convulsion is the initial symptom, hemiparesis may be considered as Todd's palsy (5). This assumption may delay the diagnosis and treatment of acute BAO, and may directly affect prognosis (14, 18). Therefore, when dealing with patients with acute convulsions in the emergency department, the existence of sustained neurological signs should be evaluated. If possible, cranial computed tomography angiography should be performed to assess the possibility of stroke, especially acute BAO. This fatal condition may be treated if diagnosed in time.

The pathophysiology and mechanism of such convulsive movements in patients with posterior circulation stroke are unclear. Traditionally, when explaining the generation of seizures, the leading role is often given to the cerebral cortex. Nonetheless, Penfield et al. postulated a “centrencephalic system” to characterize a group of neurons located in the brainstem reticular formation that functioned as a pacemaker for seizures (4, 19, 20). In rat and cat models, stimulation of the reticular formation of the midbrain, pontine, or medulla results in tonic activity followed by short-lasting clonic jerks (4). However, this theory is still controversial. Other studies have proposed that the disruption of inhibitory projections from the cortex to the brainstem can result in decerebrate posturing (1, 21). These decerebrate spasms tend to be regarded as convulsive seizures. Ischemia of the descending pathways, including the corticospinal, vestibulospinal, and reticulospinal tracts, may be related (21, 22). Zhang et al. recorded epileptic discharges from the brainstem, but not from the hippocampus or cortex, even in middle cerebral artery occlusion induced convulsive seizures (1). Mader et al. reported a patient with bilateral paramedian thalamic infarcts and BAO, followed by paramedian midbrain infarcts 12 days later, who experienced seizures witnessed by caregivers and recorded by EEG (1). Such evidence suggests the role of the brainstem in generating convulsive movements or seizures. However, the posterior circulation also provides blood supply to the occipital and temporal lobes and thalamus. A study on seizures and epilepsy in patients with a posterior circulation infarct demonstrated that > 50% of patients with seizures had infarcts in the territory of the posterior cerebral artery, which supplies the medial surface of the occipital and temporal lobes (3). Therefore, ruling out the role of the occipital and temporal lobes or thalamus in generating such convulsive movements in BAO is difficult (5, 6, 8).

In animal models, stimulation of the brain stem produces tonic-clonic activities without concurrent cortical discharges classically recorded with cortical seizures on scalp EEG (23). The EEG results available in our study demonstrated no epileptic activity, except in one patient, showing epileptic discharge from the left frontotemporal region. This might, in part, be explained that postictal or interictal EEG could be normal in this scenario. Another explanation is that scalp EEG may not demonstrate electric activity from the brainstem or medial temporal lobe. In summary, it is still controversial whether these convulsive movements are seizures or seizure mimics.

In general, the most common mechanism of BAO is *in situ* atherosclerosis. However, most cases of acute BAO with convulsions on presentation were related to embolism or vascular dissection; they were rarely related to atherosclerosis. According to the results of some clinical and autopsy studies, seizures are more common with cardioembolic infarct than other subtypes of ischemic stroke; however, clinical data showing a clear relationship between embolism and seizures are lacking (17). Embolism or vascular dissection may cause sudden ischemia and subsequent pathophysiological changes to more brain structures without providing sufficient time for collateral circulation to compensate. Posterior circulation strokes caused by atherosclerosis may also present with convulsions (24).

Poor prognosis has been reported in > 80% of patients with acute BAO (12, 13). Since 2015, several large randomized clinical trials have demonstrated the safety and efficacy of EVT in large vessel occlusions in the anterior circulation. However, it is uncertain whether patients with large vessel occlusions in the posterior circulation would also benefit from EVT. Several studies have demonstrated improvement in mortality and disability rates in patients with acute BAO receiving endovascular treatment (14, 25, 26). However, the current data are insufficient for generating high-class evidence-based guidelines. EVT, especially combined with intravenous thrombolysis, if possible, seems to be the most effective therapy for the treatment of acute BAO. Studies have also suggested that EVT within the early therapeutic time window is associated with better functional outcomes and reduced mortality (14, 26). Therefore, in patients with acute BAO presenting with convulsive movements, prompt recognition and diagnosis are crucial.

The limitations of the present study include the restricted number of patients and the incomplete availability of clinical data for some patients. Only articles published in English during the last 20 years were included. EEG results were not available for most patients. In addition, it was unclear whether some available EEG results were ictal or postictal. Moreover, due to reperfusion therapies in the hyper-acute stage, the final stroke regions demonstrated in the following diffusion-weighted imaging may be inconsistent with ischemic regions during disease onset. Therefore, it is difficult to attribute the origin of the convulsion to the brainstem, thalami, or occipital and temporal lobes.

## Conclusion

Acute BAO, especially that related to embolism or vessel dissection, can present with convulsive movements. The pathophysiology and underlying mechanisms are unclear. Therefore, defining this phenomenon as a seizure or seizure mimic is difficult. However, acute BAO is a devastating, but treatable disease. The possibility of acute BAO in patients with acute-onset convulsive movements should be considered.

## Data Availability Statement

The original contributions presented in the study are included in the article/supplementary material, further inquiries can be directed to the corresponding author/s.

## Author Contributions

DZ collected relative data and drafted the manuscript. YC and YH helped with data collection and direct patient care. XHu and XHe revised the manuscript for intellectual content. All authors contributed to the article and approved the submitted version.

## Funding

This work was supported by the Health Commission of Zhejiang Province, Medical and Health Project (2021KY727), and Hangzhou Municipal Health Commission, Medical and Health Science Project (A20210510).

## Conflict of Interest

The authors declare that the research was conducted in the absence of any commercial or financial relationships that could be construed as a potential conflict of interest.

## Publisher's Note

All claims expressed in this article are solely those of the authors and do not necessarily represent those of their affiliated organizations, or those of the publisher, the editors and the reviewers. Any product that may be evaluated in this article, or claim that may be made by its manufacturer, is not guaranteed or endorsed by the publisher.
